# Adults have moderate-to-good insight into their face recognition ability: Further validation of the 20-item Prosopagnosia Index in a Portuguese sample

**DOI:** 10.1177/1747021818765652

**Published:** 2018-05-07

**Authors:** Paulo Ventura, Lucy Anne Livingston, Punit Shah

**Affiliations:** 1 Faculty of Psychology, University of Lisbon, Lisboa, Portugal; 2 Social, Genetic & Developmental Psychiatry Centre, Institute of Psychiatry, Psychology & Neuroscience, King’s College London, London, UK; 3 Department of Psychology, University of Bath, Bath, UK

There is growing debate about whether people have insight into their face recognition ability, including a recent exchange in the *The Quarterly Journal of Experimental Psychology* (Livingston & Shah, 2017; Palermo et al., 2017). This focussed on reports that people have enough insight into their face recognition ability to justify the use of a self-report questionnaire to identify people with face recognition difficulties, for example, those with developmental prosopagnosia (DP). Shah, Gaule, Sowden, Bird, and Cook (2015) published the 20-item prosopagnosia index (PI20), a self-report questionnaire for measuring prosopagnosic traits. PI20 scores distinguish suspected developmental prosopagnosic from typically developing adults, and they correlate with behavioural measures of familiar (Famous Face Recognition Test; FFRT) and unfamiliar (Cambridge Face Memory Test; CFMT, Duchaine & Nakayama, 2006) face recognition abilities. The PI20 was further validated against a measure of face-matching ability (Glasgow Face Matching Test; Burton, White, & McNeill, 2010) that is more representative of applied settings (Shah, Sowden, Gaule, Catmur, & Bird, 2015). Turano and colleagues (Turano, Marzi, & Viggiano, 2016; Turano & Viggiano, 2017) have since developed the Italian Face Ability Questionnaire, which successfully measures individual differences in face recognition ability in Italian samples (Turano et al., 2016; Turano & Viggiano, 2017).

Palermo et al. (2017), however, argued that although individuals with DP might have relatively good insight into their face recognition abilities, due to the severity of their difficulties, typical perceivers have minimal insight (see also, Bobak, Pampoulov, & Bate, 2016). To explain the difference between their findings and those reported in Shah, Gaule, et al. (2015), Palermo et al. (2017) suggested that Shah, Gaule, et al.’s analyses, combining people with and without DP, had inflated the strength of the correlations between PI20 scores and performance on behavioural tasks. They also speculated that people with DP might have been involved in previous research and had therefore received feedback from formal testing prior to administration of the PI20. However, since Palermo et al.’s publication, Gray, Bird, and Cook (2017) have reported correlations between the PI20 scores and CFMT performance in participants that have never received feedback about their face recognition ability. Most recently, Livingston and Shah (2017) re-examined Shah, Gaule, et al.’s (2015) data, which found correlations between the PI20 and the CFMT separately in groups with and without DP. Together, converging evidence indicates that previous findings of a relationship between questionnaire and behavioural measures of face recognition are robust and unlikely to be a statistical artefact. Equally, however, Livingston and Shah (2017) re-examined, rather than replicated, data from a small sample, therefore it would be valuable to replicate these findings in a larger sample of adults. Moreover, they noted that the extent to which humans have “good” insight into their face recognition ability remains debatable and warrants further investigation.

We therefore conducted a study to advance this debate on self-reported face recognition ability. We recruited 123 participants (15 Male, *M*_age_ = 20.40 years, *SD*_age_ = 4.35) from a Portuguese University, who gave informed consent and agreed to participate in exchange for course credit. We adapted the PI20 for a Portuguese population (PI20-Portuguese; see Supplementary Material) and validated it against behavioural tasks, presented in Portuguese, measuring familiar (FFRT) and unfamiliar (CFMT) face recognition. The FFRT comprised 34 international, including four Portuguese, celebrities (actors, politicians, singers and sports people), to measure familiar face recognition. Participants had to identify the celebrities from cropped photographic images by providing their name or other identifying information. The colour images were presented in the centre of the screen on each trial and remained visible until participants responded. The FFRT had good internal consistency (Cronbach’s α = .89) and FFRT scores were calculated as a percentage of correct identifications of celebrities each participant was familiar with. Performance on this test (*M* = 71.72%, *SD* = 17.24%) was in line with previous data (e.g., Shah, Gaule, et al., 2015). The CFMT requires the recognition of six newly learnt unfamiliar faces in three stages; recognition of the same images (introduction), recognition of the same faces in different perspectives, and recognition of the same faces in different perspectives with the addition of visual noise. The trials consisted of three-alternative forced choice tests, and CFMT scores were converted to percentage accuracy (*M* = 86.20%, *SD* = 10.24%). Analyses showed that the PI20-Portuguese has a unifactorial structure and good internal consistency (α = .84). The average PI20 score, and distribution of scores (*M* = 42.02, *SD* = 9.26), was almost identical to previous results (e.g., Shah, Sowden et al., 2015). Importantly, PI20 scores were significantly correlated with the FFRT (*r* = −.39, *p* < .0001) and the CFMT (*r* = −.43, *p* < .0001), and this pattern of results (Figure 1) held after controlling for participant age and gender (FFRT: *r* = –.37, *p* < .0001; CFMT: *r* = –.43, *p* < .0001).

**Figure 1.**
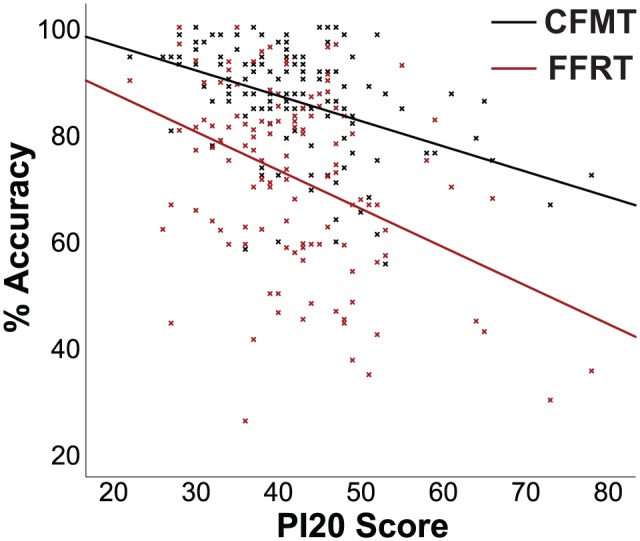
The significant correlations between PI20-Portuguese scores and performance on the Famous Faces Recognition Test (FFRT; *r* = –.39, *p* < .0001) and the Cambridge Face Memory Test (CFMT; *r* = –.43, *p* < .0001).

These findings provide further evidence that adults have insight into their face recognition ability, in line with the recent research on this topic (see Livingston & Shah, 2017). Encouragingly, this finding has now been reported in several studies using questionnaire measures in different languages (English, Italian, and now Portuguese). In addition, the moderate-to-large size of the relationship between questionnaire and behavioural measures of face recognition (~*r* = .40) is now consistently being found across studies. Interestingly, these recent results, including this study, sit in between Shah, Gaule, et al.’s (2015) claim that adults have “good insight” and Palermo et al.’s (2017) argument that adults “lack insight,” providing strong indication that adults have *moderate-to-good* insight into their face recognition ability.

Overall, numerous strands of evidence suggest that although traditional behavioural testing remains a more precise way to measure face recognition ability, well-validated self-report questionnaires are useful research (and potentially clinical) tools. It is hoped that the results of this study help move academic debate on from whether or not to use questionnaire measures of face recognition, particularly in studies on prosopagnosia (Shah, 2016), towards refining and improving these instruments to better understand the psychological causes and consequences of (a)typical face recognition ability. More generally, the Portuguese version of the PI20 reported in this study could be used in future research in Portuguese-speaking countries (e.g., Brazil), hopefully providing opportunities to advance (cross-cultural) face recognition research in new and diverse samples across the population.

## Supplementary Material

Supplementary material

Supplementary material
