# Supplementary material for: Adults have moderate-to-good insight into their face recognition ability: Further validation of the 20-item Prosopagnosia Index in a Portuguese sample
Source: Q J Exp Psychol (Hove). 2018 May 7;71(12):2677–9. doi: 10.1177/1747021818765652 (PMC6293437; doi:10.1177/1747021818765652)
Supplement: Supplementary material [file Supplemental_Material_Ventura_et_al.pdf]

Supplemental Material - Portuguese 20-Item Prosopagnosia Index (PI20-Portuguese)[1]

1. **A minha capacidade de reconhecimento facial é pior do que a da maioria das pessoas**

Concordo fortemente ❑ Concordo ❑ Não concordo, nem discordo ❑ Discordo ❑ Discordo fortemente ❑

2. **Eu sempre tive uma má memória para os rostos**

Concordo fortemente ❑ Concordo ❑ Não concordo, nem discordo ❑ Discordo ❑ Discordo fortemente ❑

3. **Considero especialmente mais fácil reconhecer pessoas que têm características faciais distintivas**

Concordo fortemente ❑ Concordo ❑ Não concordo, nem discordo ❑ Discordo ❑ Discordo fortemente ❑

4. **Muitas vezes confundo pessoas que conheci antes com estranhos**

Concordo fortemente ❑ Concordo ❑ Não concordo, nem discordo ❑ Discordo ❑ Discordo fortemente ❑

5. **Quando eu estava na escola, debatia-me para reconhecer os meus colegas de classe**

Concordo fortemente ❑ Concordo ❑ Não concordo, nem discordo ❑ Discordo ❑ Discordo fortemente ❑

6. **Quando as pessoas mudam o penteado ou usam chapéus, eu tenho problemas em reconhecê-las**

Concordo fortemente ❑ Concordo ❑ Não concordo, nem discordo ❑ Discordo ❑ Discordo fortemente ❑

7. **Às vezes tenho que avisar novas pessoas que conheço de que sou “mau com caras”**

Concordo fortemente ❑ Concordo ❑ Não concordo, nem discordo ❑ Discordo ❑ Discordo fortemente ❑

8. **Acho fácil imaginar rostos individuais na minha mente**

Concordo fortemente ❑ Concordo ❑ Não concordo, nem discordo ❑ Discordo ❑ Discordo fortemente ❑

9. **Sou melhor que a maioria das pessoas em colocar um "nome para um dado rosto"**

Concordo fortemente ❑ Concordo ❑ Não concordo, nem discordo ❑ Discordo ❑ Discordo fortemente ❑

10. **Sem ouvir as vozes das pessoas, debato-me para as reconhecer**

Concordo fortemente ❑ Concordo ❑ Não concordo, nem discordo ❑ Discordo ❑ Discordo fortemente ❑

---

[1] The original PI20 was translated in line with a standard ‘backward translation’ procedure. It was translated from English into Portuguese, and back into English, by three bilingual translators, including an experimenter (P.V.). Any discrepancies were then discussed with the first author of the original English PI20 (P.S.) before producing this final Portuguese version of the questionnaire.

11. **A ansiedade sobre o reconhecimento facial levou-me a evitar certas situações sociais ou profissionais**

Concordo fortemente ❑ Concordo ❑ Não concordo, nem discordo ❑ Discordo ❑ Discordo fortemente ❑

12. **Eu tenho que tentar mais do que as outras pessoas para conseguir memorizar rostos**

Concordo fortemente ❑ Concordo ❑ Não concordo, nem discordo ❑ Discordo ❑ Discordo fortemente ❑

13. **Tenho muita confiança na minha capacidade de me reconhecer em fotografias**

Concordo fortemente ❑ Concordo ❑ Não concordo, nem discordo ❑ Discordo ❑ Discordo fortemente ❑

14. **Às vezes acho os filmes difíceis de seguir por causa de dificuldades em reconhecer os personagens**

Concordo fortemente ❑ Concordo ❑ Não concordo, nem discordo ❑ Discordo ❑ Discordo fortemente ❑

15. **Os meus amigos e familiares acham que eu tenho um reconhecimento de rostos ou memória para rostos ruins**

Concordo fortemente ❑ Concordo ❑ Não concordo, nem discordo ❑ Discordo ❑ Discordo fortemente ❑

16. **Sinto que frequentemente ofendo as pessoas ao não reconhecer quem elas são**

Concordo fortemente ❑ Concordo ❑ Não concordo, nem discordo ❑ Discordo ❑ Discordo fortemente ❑

17. **É fácil para mim reconhecer indivíduos em situações que exigem que as pessoas usem roupas semelhantes (por exemplo, fatos, uniformes e roupa de natação)**

Concordo fortemente ❑ Concordo ❑ Não concordo, nem discordo ❑ Discordo ❑ Discordo fortemente ❑

18. **Em encontros familiares, às vezes confundo membros individuais da família**

Concordo fortemente ❑ Concordo ❑ Não concordo, nem discordo ❑ Discordo ❑ Discordo fortemente ❑

19. **Acho fácil reconhecer celebridades em fotos "antes de eles serem famosos", mesmo que tenham mudado consideravelmente**

Concordo fortemente ❑ Concordo ❑ Não concordo, nem discordo ❑ Discordo ❑ Discordo fortemente ❑

20. **É difícil reconhecer as pessoas familiares quando as encontro fora do contexto (por exemplo, encontrar um colega de trabalho inesperadamente enquanto estou às compras)**

Concordo fortemente ❑ Concordo ❑ Não concordo, nem discordo ❑ Discordo ❑ Discordo fortemente ❑
